# A novel approach for ASD recognition based on graph attention networks

**DOI:** 10.3389/fncom.2024.1388083

**Published:** 2024-04-10

**Authors:** Canhua Wang, Zhiyong Xiao, Yilu Xu, Qi Zhang, Jingfang Chen

**Affiliations:** ^1^School of Computer, Jiangxi University of Chinese Medicine, Nanchang, China; ^2^School of Electronic & Information Engineering, Jiangxi Institute of Economic Administrators, Nanchang, China; ^3^School of Software, Jiangxi Agricultural University, Nanchang, China; ^4^Department of Medical Imaging, Affiliated Hospital of Jiangxi University of Chinese Medicine, Nanchang, China; ^5^Department of Medical Imaging, The Second Affiliated Hospital of Nanchang University, Nanchang, China

**Keywords:** autism, graph, self-attention, classification, fMRI

## Abstract

Early detection and diagnosis of Autism Spectrum Disorder (ASD) can significantly improve the quality of life for affected individuals. Identifying ASD based on brain functional connectivity (FC) poses a challenge due to the high heterogeneity of subjects’ fMRI data in different sites. Meanwhile, deep learning algorithms show efficacy in ASD identification but lack interpretability. In this paper, a novel approach for ASD recognition is proposed based on graph attention networks. Specifically, we treat the region of interest (ROI) of the subjects as node, conduct wavelet decomposition of the BOLD signal in each ROI, extract wavelet features, and utilize them along with the mean and variance of the BOLD signal as node features, and the optimized FC matrix as the adjacency matrix, respectively. We then employ the self-attention mechanism to capture long-range dependencies among features. To enhance interpretability, the node-selection pooling layers are designed to determine the importance of ROI for prediction. The proposed framework are applied to fMRI data of children (younger than 12 years old) from the Autism Brain Imaging Data Exchange datasets. Promising results demonstrate superior performance compared to recent similar studies. The obtained ROI detection results exhibit high correspondence with previous studies and offer good interpretability.

## 1 Introduction

Autism spectrum disorder (ASD) is a complex neuro-developmental disorder that impairs social communication, language skills, and behavior (Wise et al., 2015; Takayanagi et al., 2022). Recent estimates from the Centers for Disease Control and Prevention suggest that approximately 1 in 36 children grapples with ASD. Presently, the etiology and pathogenesis of ASD remain elusive, and identification and diagnosis rely on simplistic symptomatic observation and empirical judgment by clinicians (Sauer et al., 2021; Klin, 2022; Zahra et al., 2022; Majhi et al., 2023). Artificial intelligence-assisted diagnosis of ASD can alleviate the contradiction between supply and demand between limited psychiatrists and more ASD patients (Abbas et al., 2020; Mertz, 2021). Early detection and intervention for ASD can enhance language, social, and learning skills in affected children, concurrently fostering optimal brain development (Bejarano-Martin et al., 2020; Dai et al., 2023).

Neuroimaging, particularly functional magnetic resonance imaging (fMRI), stands out as a crucial tool for unraveling the intricate neural underpinnings of ASD (Nijhof et al., 2018; Noriega, 2019). Numerous machine learning (ML) and deep learning (DL) algorithms have been proposed for the identification of ASD based on fMRI datasets (Wang et al., 2019; Almuqhim and Saeed, 2021; Khodatars et al., 2021). For instance, Bi et al. used a random SVM cluster to classify 45 ASD and 39 typical development (TD), with an accuracy of 96.15% (Bi et al., 2018). Wee et al. employed a multi-kernel SVM to classify 58 ASD and 59 TD cases, achieving 96.27% accuracy (Wee et al., 2014). It’s crucial to note that these studies based on ML often suffered from limitations. The subjects were frequently sourced from a single research institution, leading to small sample sizes. For instance, one study included only 13 subjects with ASD and 14 subjects with TD (Murdaugh et al., 2012). To surmount these limitations, efforts have been directed toward deploying DL methods with larger-scale datasets. For instance, Aghdam et al. achieved a classification accuracy of 65.56% utilizing deep belief networks (Akhavan Aghdam et al., 2018). Similarly, Heinsfeld et al. obtained a classification accuracy of 70% using an auto-encoder based on the ABIDE datasets (Heinsfeld et al., 2018). Ma et al. achieved a notable 74.73% accuracy in distinguishing ASD patients from 306 ASD and 341 TD (Ma et al., 2023).

The algorithms mentioned above, whether utilizing ML or DL methods, have made meaningful explorations in identifying ASD. However, challenges arise when extending these models to larger population samples across different sites. One the one hand, the accuracy of ML models in identifying ASD tends to significantly decrease when applied to a completely new datasets. This is mainly due to the heterogeneity of fMRI data, which arises from differences in equipment, parameters, ethnicity, etc., across multiple sites collecting fMRI data. One the other hand, while DL models can mitigate the interference caused by this heterogeneity, the models often lack interpretability.

To address these challenges, some ASD identification methods based on graph neural networks (GNN) have emerged, which can provide good interpretability. The key to the method of using GNN is how to construct the graph. For example, Li et al. proposed an interpretable GNN called BrainGNN (Li et al., 2021); Wen et al. proposed a multi-view graph convolution networks (MVS-GCN) for ASD diagnosis (Wen et al., 2022); The BrainGNN model and MVS-GCN model have many parameters, long training time, and the classification accuracy needs to be further improved. It is worth noting that not all GNN-based methods have good interpretability, for example, the relational graph attention networks (RGAT) proposed by Gu et al., which treats each subject as a node of a graph and cannot mine abnormal brain regions (Gu et al., 2023).

For the above mentioned issues in ASD recognition, our paper proposes a pioneering approach for ASD recognition based on graph attention networks. The novel method strategically treats the region of interest (ROI) within subjects’ brains as fundamental node in a graph representation. This graph-based approach enables a more transparent and interpretable representation of the complex relationships within the data. Leveraging the connectivity structure encoded by the optimized functional connectivity (FC) as the adjacency matrix, we aim to provide a holistic view of the interplay between brain regions in individuals with ASD. The inclusion of the self-attention mechanism further enhances the model’s ability to capture nuanced dependencies within the data, addressing a current limitation in the interpretability of existing models. Recognizing the necessity for interpretability in ASD identification, our framework incorporates node-selection pooling layers. These layers play a crucial role in determining the importance of individual ROI, thereby offering a clear rationale for the model’s predictions.

To validate the effectiveness of our proposed framework, we apply it to fMRI data collected from children aged below 12 years, obtained from the Autism Brain Imaging Data Exchange (ABIDE) datasets. The outcomes of our experiments reveal promising results, showcasing superior performance compared to recent studies. Additionally, the obtained ROI detection results exhibit a high level of correspondence with findings from previous studies, further reinforcing the robustness and interpretability of our proposed approach.

In the subsequent sections, we delve into the methodology, experimental setup, and results, providing a comprehensive exploration of our innovative approach and its contributions to the field of ASD identification. Through this research, we aim to bridge the existing gap between advanced machine learning techniques and clinical interpretability, fostering a more effective and practical approach to ASD detection and diagnosis.

## 2 Materials and methods

### 2.1 Participants and data preprocessing

This study specifically targets children aged 12 years old or younger. We employ the ABIDE datasets and adhere to specific criteria in selecting research subjects: (1) Participants are children aged 12 years old or younger; (2) Each site contributes no fewer than 40 subjects; (3) The ratio of ASD to TD subjects at each site is approximately equal. Consequently, a total of 264 subjects (134 ASD and 130 TD) from 5 sites have been selected. The details of these subjects are shown in Table 1.

**Table 1:** Scanning parameters and subjects in different sites.

Site	MRI vendor	TR (msec.)	ASD	TD
NYU	Siemens	2000	43	41
UM	GE	2000	23	23
KKI	Phillips	2,500	22	22
UCLA	Siemens	3,000	26	24
STAN	GE	2000	20	20

NYU, Langone Medical Center; UM, University of Michigan; KKI, Kennedy Krieger Institute; UCLA, University of California Los Angeles; STAN, Stanford University, TR, repetition time.

All fMRI data preprocessing was conducted using the DPARSF software (Chao-Gan and Yu-Feng, 2010), following these specific steps: (1) Exclusion of the first 10 time points, (2) slice timing correction, (3) head motion realignment, (4) registration of individual structural T1-weighted images to the mean functional images using a 6-degree-of-freedom linear transformation, (5) segmentation, (6) nuisance covariate regression (GSR was not performed due to concerns about increasing negative correlations), (7) normalization using DART- EL, and (8) temporal filtering.

### 2.2 Construction of the brain graph

The brain graph is a crucial component of the proposed framework, as it represents the connectivity patterns among various brain regions. The brain is segmented into 200 ROIs using the CC200 Atlas (Craddock et al., 2012). These ROIs are defined as graph nodes $V=\left\{v_{1},v_{2},\ldots ,v_{200}\right\}$. An undirected weighted graph is represented as $G=\left(V,E\right)$, where Eis the edge set, i.e., a collection of $\left(v_{i},v_{j}\right)$ linking vertices from $v_{i}$ to $v_{j}$, and is derived from the brain FC network. In addition, *G* includes an associated node feature set $H=\left\{h_{1},h_{2},\ldots ,h_{200}\right\}$, such as $h_{2}$ is the feature vector associated with node $v_{2}$.

#### 2.2.1 Construction of the node feature

To extract node features, we conduct wavelet transformations on the BOLD signals within the ROI. Specifically, if the BOLD signal in a given ROI is denoted as $x_{i}$, the Daubechies wavelet transformation is utilized to process $x_{i}$and decompose it into 6 layers, with ‘db1’ being employed as the wavelet basis function throughout the transformation. The decomposition yields approxim- ation coefficients at level 6 and detailed coefficients spanning levels 1 through 6. The expression for the transformation is as follows:


(1) $$\mathrm{coeffs}\left[i\right]=\left[cA6^{i},cD6^{i},cD5^{i},cD4^{i},cD3^{i},cD2^{i},cD1^{i}\right]$$

here, $cA6^{i}$ represents the approximation coefficients at level 6, and $cD6^{i}$, $cD5^{i}$, $cD4^{i}$, $cD3^{i}$, $cD2^{i}$, $cD1^{i}$ represent the detail coefficients at levels 6 to 1.

**Figure 1:**
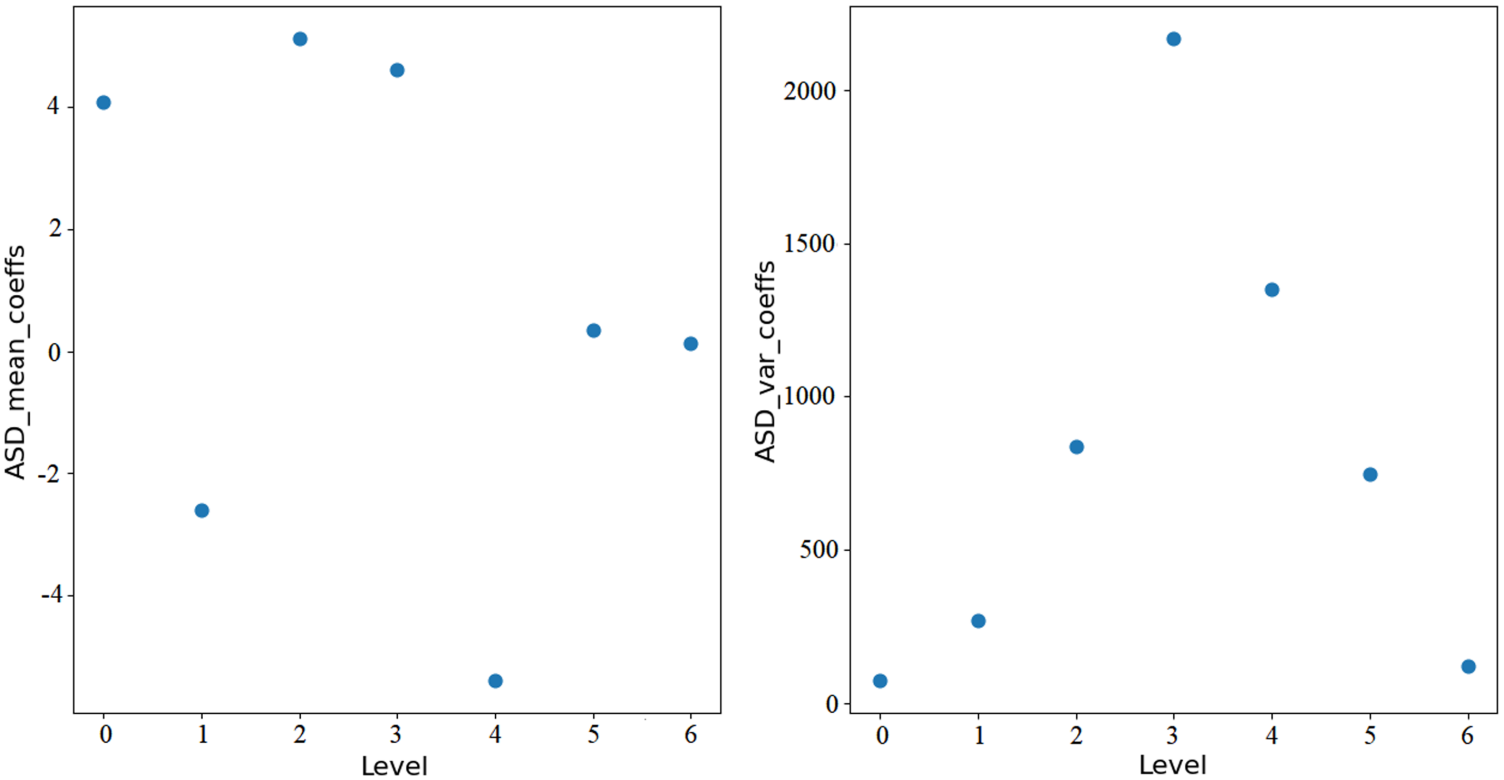
Wavelet features of ASD.

**Figure 2:**
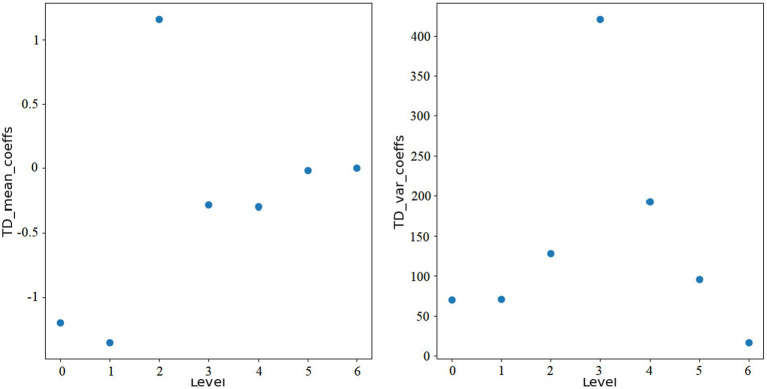
Wavelet features of TD.

Then, we use the following formula to calculate the mean and variance of each level of wavelet coefficients.


(2) $$\mathrm{mean}:\mathrm{coeffs}\left[i\right]=\frac{1}{N}\overset{N-1}{\underset{j=0}{\sum }}\mathrm{coeffs}\left[i\right]$$

(3) $$\mathrm{var}:\mathrm{coeffs}\left[i\right]=\frac{1}{N}\overset{N-1}{\underset{j=0}{\sum }}{\left(\mathrm{coeffs}\left[i\right]-\mathrm{mean}:\mathrm{coeffs}\left[i\right]\right)}^{2}$$

here, *i* represents the level of wavelet transform, and *N* is the length of each wavelet coefficient array. Figures 1, 2 show the features extracted from the same ROI of any ASD and TD using wavelet transform, respectively.

Through wavelet transformation, the persistent patterns in brain activity (corresponding to approximation coefficients) and transient changes (corresponding to detail coefficients) are extracted. Compared to traditional node feature representations, wavelet transformation can extract features that are more discriminative. Ultimately, we integrate the *mean_coeffs* and *var_coeffs* with the mean and variance of the BOLD signal itself to compose the node’s feature. Hence, node feature $h_{i}\in R^{\left(16\right)}$.

#### 2.2.2 Construction of the adjacency matrix

We utilize the optimized brain FC network to build the adjacency matrix. Initially, the Pearson correlation coefficient between any two nodes’ time series is calculated to derive the FC matrix using the subsequent formula:


(4) $$E\left(i,j\right)=\frac{\overset{N-1}{\underset{n=0}{\sum }}{\left(x_{\mathrm{in}}-{\bar{x}}_{i}\right)}^{T}\left(x_{jn}-{\bar{x}}_{j}\right)}{\sqrt{\overset{N-1}{\underset{n=0}{\sum }}{\left(x_{\mathrm{in}}-{\bar{x}}_{i}\right)}^{2}}\sqrt{\overset{N-1}{\underset{n=0}{\sum }}{\left(x_{jn}-{\bar{x}}_{j}\right)}^{2}}}$$

where, $x_{\mathrm{in}}$, $x_{jn}$, ${\bar{x}}_{i}$ and ${\bar{x}}_{j}$denote the time-courses of node *i*, time-course of node *j* at time point *n*, the mean of the time-courses of node *i* and the mean of time-courses of node *j, respectively. N* represents the length of the BOLD signal.

To address the interference stemming from heterogeneity in multi-site fMRI data, some measures were adopted: on one hand, the FC matrices of all ASD and TD subjects were averaged separately; on the other hand, a predefined threshold was used to filter out unimportant connections, meaning that elements in the averaged FC matrices above the threshold were preserved while those below the threshold were set to zero. The FC matrices optimized through the above steps were used as adjacency matrices. Additionally, the consistency and comparability of the data were also enhanced to some extent by preprocessing all fMRI data using the same steps, as described previously.

### 2.3 A model for ASD classification based on graph attention networks (GAT)

**Figure 3:**
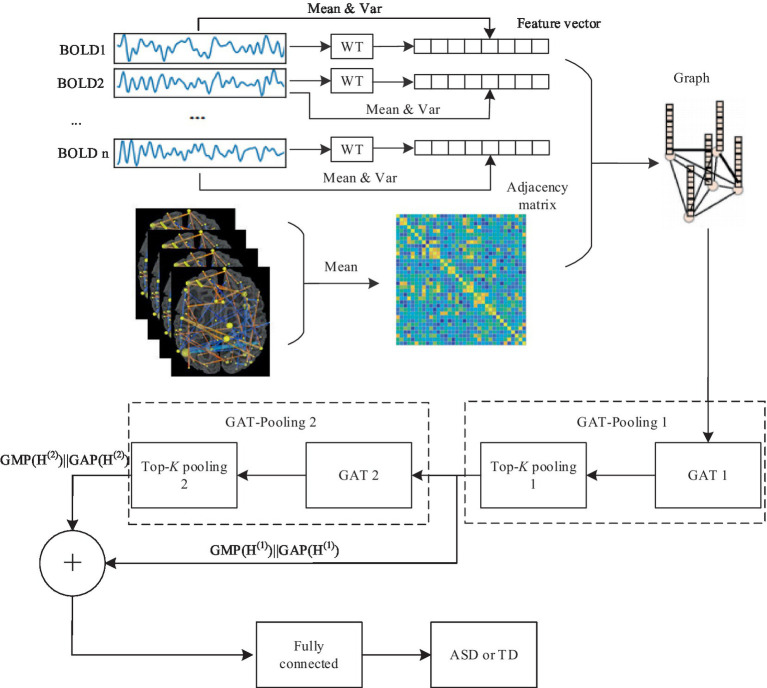
The overview of ASD prediction pipeline.

The GAT-based model comprises three types of layers: GAT layers, top-*K* pooling layers, and fully connected layers. The model architecture is depicted in Figure 3.

#### 2.3.1 GAT layer

The GAT employs attention mechanism which assigns different weights to different neighbors based on their relevance to the node being encoded (Wan et al., 2024). Let the $i^{th}$ node features in the $l^{th}$ layer be $h_{i}^{\left(l\right)}\in R^{d^{\left(l\right)}}$, the GAT operation can be expressed as follows:


(5) $$h_{i}^{\left(l\right)}=\sigma \left(\underset{j\in \mathrm{Neighbors}\left(i\right)}{\sum }\alpha_{ij}W^{\left(l-1\right)}h_{j}^{\left(l-1\right)}\right)$$

here, σis the activation function, typically ReLU, $W^{\left(l-1\right)}$is a learnable weight matrix, $\alpha_{ij}$represents attention coefficients and $h_{j}^{\left(l-1\right)}$denotes the input features of neighboring node j.

In formula (5), the calculation of attention coefficients $\alpha_{ij}$is a crucial aspect. We utilize multiple attention heads can capture different aspects of relationships. After obtaining the attention coefficients from each attention head, these coefficients are aggregated by taking an average to form the final attention coefficients for each node. The attention coefficient $\alpha_{ij}^{k}$ in the *k-th* attention head is as follows:


(6) $$\alpha_{ij}^{k}=\frac{\exp\left(\alpha_{k}^{T}\left[W_{k}h_{i}\|W_{k}h_{j}\right]\right)}{\sum_{k\in \mathrm{Neighbors}\left(i\right)}\exp\left(\alpha_{k}^{T}\left[W_{k}h_{i}\|W_{k}h_{k}\right]\right)}$$

here, $\alpha_{k}$is a learnable parameter vector for the *k-th* attention head, ∥ denotes concatenation, $W_{k}$is learnable weight matrices.

In addition, we added L2 regularization to the GAT layers to avoid overfitting. The regularization parameter for this process is denoted as *weight_decay*.

#### 2.3.2 Top-*K* pooling layer

The top-*K* pooling layer is a pooling operation that selects the top K nodes based on attention scores. For every edge in the graph, we arrange the nodes according to their combined attention scores and then choose the top K nodes. Specifically, the process can be outlined as follows:

Step 1: Node-level aggregation.

For each node *i*, aggregate the attention scores across its incoming edges. This could involve summing or averaging the attention scores:


(7) $$Node:\mathrm{attentio}n_{i}=\underset{j\in \mathrm{Neighbors}\left(i\right)}{\sum }\alpha_{ij}$$

Step 2: Global aggregation.

Aggregate the aggregated attention scores across all nodes in the graph. This results in a global attention score for each node, representing its overall importance:


(8) $$\mathrm{Global}:\mathrm{attentio}n_{i}=\underset{i\in \mathrm{Nodes}}{\sum }\mathrm{Node}:\mathrm{attentio}n_{i}$$

Step 3: Top-*K* selection:

Sort the nodes based on their global attention scores in descending order and select the top K nodes with the highest global attention scores. Mathematically, the selection process can be represented as:


(9) $$Sele\mathrm{cted}:\mathrm{nodes}=\mathrm{Top}-K\left(\mathrm{Nodes},\mathrm{Global}:\mathrm{attentio}n_{i}\right)$$

#### 2.3.3 Fully connected layer

The fully connected layers play a important role in consolidating multi-scale features derived from the GAT-Pooling 1 and GAT-Pooling 2 block (Chen et al., 2023; Li et al., 2023; Wan et al., 2023a). We feed the multi-scale features $Z^{\left(3\right)}$ to the fully connected layer. $Z^{\left(3\right)}$ is written as:


(10) $$Z^{\left(3\right)}=Z^{\left(1\right)}+Z^{\left(2\right)}$$

here, $Z^{\left(1\right)}$_and_ $Z^{\left(2\right)}$ are the output which underwent Global Maximum Pooling (GMP) and Global Average Pooling (GAP) from the GAT-Pooling 1 and GAT-Pooling 2 block, respectively. $Z^{\left(1\right)}$_and_ $Z^{\left(2\right)}$ can be represented as:


(11) $$Z^{\left(1\right)}=GMP\left(H^{\left(1\right)}\right)\|\mathrm{GAP}\left(H^{\left(1\right)}\right)$$

(12) $$Z^{\left(2\right)}=GMP\left(H^{\left(2\right)}\right)\|\mathrm{GAP}\left(H^{\left(2\right)}\right)$$

Lastly, batch normalization is applied for enhanced training stability, and dropout is utilized for regularization. The final linear layer employs a sigmoid activation function, compressing the output to a one-dimensional tensor, suitable for the binary classification task of ASD identification.

## 3 Results and discussion

The model incorporated GAT layers and top-*k* pooling to capture graph-based features at different scales. It was trained with the cross-entropy loss function and optimized using the Adam optimizer. The model was instantiated with input features of size 16, GAT 1 and GAT 2 sizes of 32 each, and utilized 4 attention heads. The proposed algorithm was performed using the HP personal computer (CPU: Intel core i7 2.6 GHz; RAM: 16 GB; NVIDIA Quadro P600: 4GB) and Torch-1.8.0 + cu111. The classification quality was assessed by the following performance indices:


(13) $$\mathrm{Accuracy}=\left(TP+TN\right)/\left(TP+FN+TN+FP\right)$$

(14) $$\mathrm{Sensitivity}=TP/\left(TP+FN\right)$$

(15) $$\mathrm{Specificity}=TN/\left(TN+FP\right)$$

Here, *TP*, *FN*, *TN*, and *FP* denote, respectively, the number of ASD correctly classified, the number of ASD predicted to be TD, the number of TD correctly classified, and the number of TD predicted to be ASD.

### 3.1 Result of single site

In the proposed model, the *weight_decay* was set to 0.0001, the ratio of the top_*k* pooling was set to 0.2, the dropout rate was set to 0.5, the learning rate was set to 0.001 and the number of epochs was 30. As shown in Table 2, our proposed algorithm for childhood ASD achieved promising results across different sites by the 5-fold cross-validation strategy.

**Table 2:** Results of classification at different sites.

Site	Accuracy	Sensitivity	Specificity
NYU	76.47%	66.67%	87.50%
UM	83.33%	77.50%	90.00%
KKI	76.39%	75.00%	77.50%
UCLA	70.00%	66.67%	75.00%
STAN	87.50%	83.33%	100%

The algorithm at the NYU site achieved a moderate accuracy of 76.47%. It demonstrated higher specificity (87.50%) than sensitivity (66.67%), indicating a better ability to correctly identify TD cases. At the UM site, the algorithm showcased a relatively high accuracy of 83.33%. The site exhibited balanced sensitivity (77.50%) and specificity (90.00%), suggesting a good overall performance in correctly identifying both ASD and TD cases. The KKI site displayed a moderate accuracy of 76.39%. Similar sensitivity (75.00%) and specificity (77.50%) were observed, suggesting a balanced performance but with room for improvement. The STAN site showcased the highest accuracy of 87.70%. High specificity (100%) indicates an excellent ability to identify TD cases, although sensitivity (83.33%) could be further improved.

The algorithm’s effectiveness varies across sites, emphasizing the need for site-specific adjustments or considerations in its application. High specificity is crucial to avoid false positives, ensuring accurate identification of TD cases. Further refinement may be needed, especially in achieving higher sensitivity for enhanced ASD case detection. In summary, the algorithm exhibits promising performance but also highlights the importance of considering site-specific factors and continuous refinement for optimal results in childhood ASD identification.

### 3.2 Result of multiple sites

To further evaluate the performance of the proposed model, we conducted experiments on a multi-site fMRI datasets with 134 ASD subjects and 130 TD subjects. It was worth mentioning that, in order to overcome the interference caused by the heterogeneity of multiple sites data, we averaged the FC matrix mean of all ASD and TD subjects, respectively, and zero the connection coefficient with values less than 0.4. The *weight_decay* was set to 0.001, the ratio of the top_*k* pooling was set to 0.2, the dropout rate was set to 0.5, the learning rate was set to 0.001 and the number of epochs was 100. The proposed algorithm achieved an accuracy of 74.07%, a sensitivity of 69.23% and specificity of 78.57% by the 10-fold cross-validation.

We conducted a comparative analysis between the proposed algorithm model and several recent graph neural network-based models. To ensure fairness, we utilized the same datasets and employed 10-fold cross-validation for evaluation. The results of the comparative analysis are presented in Table 3.

**Table 3:** Results of classification with different methods.

Model	Accuracy	Sensitivity	Specificity
BrainGNN	66.67%	69.23%	64.29%
MVS-GCN	70.37%	65.69%	75.09%
RGAT	69.23%	66.67%	71.43%
The proposed algorithm	74.07%	69.23%	78.57%

The BrainGNN model achieved a moderate accuracy of 66.67%. Its balanced sensitivity (69.23%) and specificity (64.29%) suggest a fair ability to identify both ASD and TD cases. The MVS-GCN model demonstrated a relatively higher accuracy of 70.37%. Its balanced sensitivity (65.69%) and notably higher specificity (75.09%) suggest a robust overall performance. The RGAT model showcased a competitive accuracy of 69.23%, displaying a sensitivity of 66.67% and specificity of 71.43%. The proposed algorithm not only achieves a higher accuracy but also demonstrates a remarkable specificity, indicating its effectiveness in correctly identifying TD cases. This superior performance positions the proposed algorithm as a robust and promising approach for ASD classification compared to the other models.

Our proposed algorithm exhibits superiority for several key reasons. Firstly, by leveraging wavelet transformation to extract temporal features from ROI time series, we effectively capture the spatiotemporal characteristics of BOLD signals, robustly constructing features for nodes in the brain graph. Secondly, we employ an averaging approach on the FC matrix and threshold elements below a predefined threshold, potentially eliminating spurious connections and enhancing the representation of genuine edges. Thirdly, the utilization of attention mechanisms enables the identification of influential connections, pinpointing nodes that play crucial roles in the network. Lastly, we adopt a multi-scale perspective by separately extracting features from two graph convolutional layers and concatenating them, providing a comprehensive representation of node features (Wan et al., 2023b,c). These combined strategies contribute to the enhanced performance of our algorithm.

### 3.3 Interpretability

The nodes and edges selected from the top-*k* pooling layer play a crucial role in identifying ASD, enhancing the model’s interpretability. By utilizing the top-*k* pooling layer, we identified key ROIs from both individual and multiple sites, as shown in Table 4. The numbering of these ROIs corresponds to the definitions in the CC200 Atlas based on the tcorr05_2level_all.nii template (Craddock et al., 2012).

**Table 4:** Results of classification at different sites.

Site	The important ROI
NYU	9, 14, 40, 42, 44, 47, 52, 63, 65, 77, 109, 110, 116, 140, 143, 144, 159, 161,173, 179, 180, 193, 194, 199
UM	32, 33, 34, 35, 36, 37, 38, 39, 40, 41, 42, 43, 44, 45, 46, 47, 57, 58, 59
KKI	5, 40, 41, 42, 43, 44, 45, 46, 47, 83, 85, 87, 90, 101, 103, 112,124, 126, 145, 183
UCLA	5, 13, 23, 32, 37, 40, 41, 42, 43, 44, 45, 46, 47, 50, 59, 64, 78, 92, 95, 108, 110, 135, 155, 196
STAN	35, 40, 41, 42, 43, 44, 45, 46, 47, 99, 105, 106, 144, 147, 164, 166, 168, 169,181, 187, 192, 197
Multiple sites	7, 36, 40, 41, 42, 43, 44, 45, 46, 47, 59, 78, 99, 115, 117, 127, 131, 142,149, 151, 153, 156, 179

**Figure 4:**
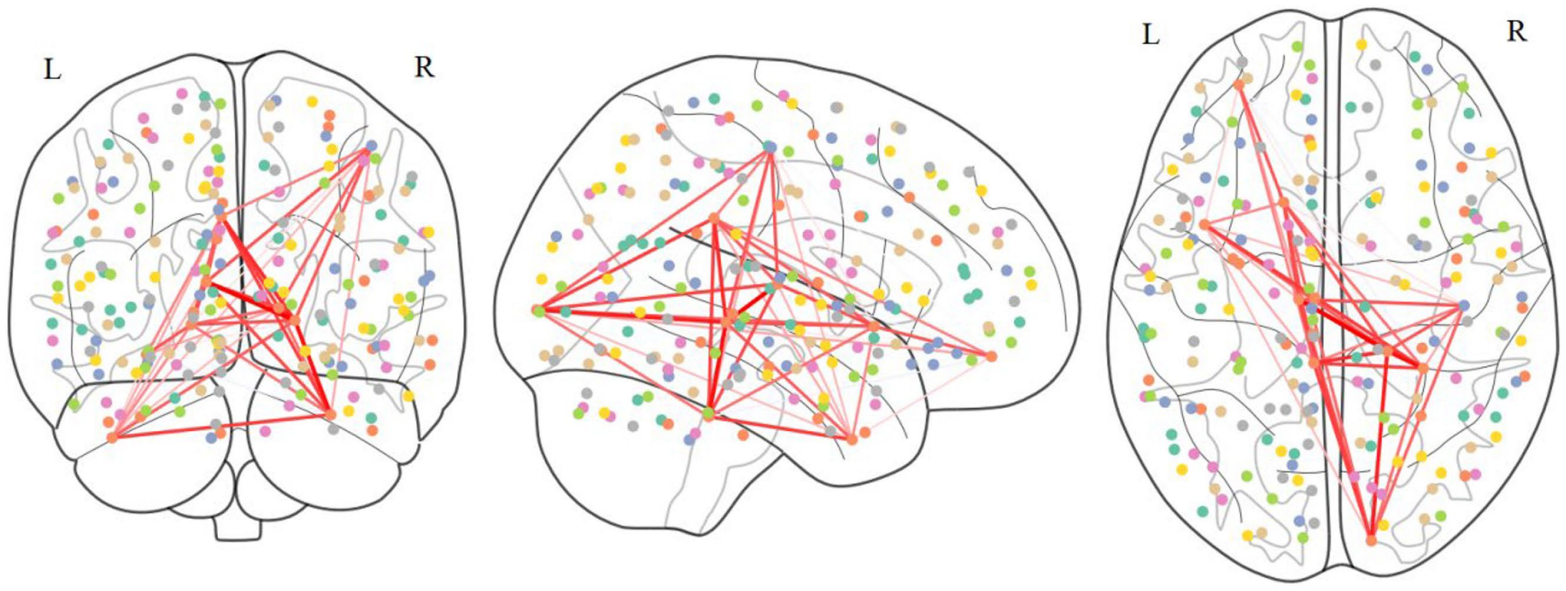
Connection patterns that are consistently present in the brains of ASD patients.

According Table 4, the distribution of important ROIs across different sites reveals interesting patterns. For instance, certain ROIs, such as ROI 40, 41, 42, 43, 44, 45, and 46, consistently appear as important across multiple sites, indicating their potential significance in ASD diagnosis irrespective of the specific population or data acquisition protocol. Conversely, some ROIs, such as ROI 35, show site-specific importance, suggesting variations in ASD-related neural substrates among different populations or data acquisition settings. These consistently appearing ROIs were mapped to the corresponding regions in the Brodmann’s brain parcellation template, as illustrated in Table 5. The connections among these ROIs are depicted in Figure 4. We proceed with a comprehensive analysis of the results to gain insights into the neural mechanisms underlying ASD and the performance of our model.

**Table 5:** Results of classification with different methods.

The most discriminative ROI	Brodmann area	Brain area
40	BA37_R	Fusiform gyrus
41	BA11_L	Orbitofrontal area
42	BA20_L	Inferior temporal gyrus
43	BA_18R	Sencondary visual cortex
44	None	Thalamus_L
45	BA23_L	Ventral posterior cingulate cortex
46	BA11_L	Orbitofrontal area
47	BA27_R	Piriform cortex
59	BA4_R	Primary motor cortex

Firstly, ROIs associated with social cognition and language processing, such as ROI 40 and 42, are consistently highlighted across various sites, aligning highly with existing literature on ASD-related alterations in social and language-related brain networks. The fusiform gyrus (ROI 40), an essential brain region for face recognition processes, has been implicated in previous studies. Task-based fMRI investigations have revealed decreased activation in the fusiform gyrus during face recognition tasks in individuals with ASD. Furthermore, research suggests that the accuracy of face recognition in ASD patients can serve as a crucial indicator predicting the severity of later symptoms. Therefore, localized abnormalities in the FC of the fusiform gyrus may be associated with difficulties in face recognition, contributing to social interaction difficulties observed in individuals with autism. The inferior temporal gyrus (ROI 40) plays a crucial role in language and visual cognition, and localized FC abnormalities in the inferior temporal gyrus may contribute to language impairments observed in individuals with ASD. Previous task-based fMRI studies have also highlighted the involvement of the inferior temporal gyrus in working memory, a domain where ASD patients often exhibit anomalies, showing correlations with repetitive behaviors. This findings strengthens the validity of our model’s predictions and underscores the importance of these brain regions in ASD pathophysiology.

Secondly, the frequency of ROIs 41, 43, 44, 45, 46, 47, and 59 is also very high. These abnormal regions can also correspond to previous research findings. For example, some studies have found that patients with autism show reduced activity in the orbitofrontal cortex (ROI 41), which may be related to their difficulties in social interaction and emotional processing. The secondary visual cortex (ROI 43) of patients with autism may be overactive, potentially connected to their heightened sensitivity to visual stimuli. Functional abnormalities in the primary motor cortex (ROI 59) might contribute to their difficulties in motor coordination and execution. Overall, these findings suggest that autism might involve abnormalities across multiple brain regions, which could underlie the condition’s symptoms related to social interaction, sensation, emotion, and movement. However, these results require further investigation for confirmation and deeper understanding.

In addition, the observation of certain ROIs being identified exclusively at specific sites warrants further investigation into potential site-specific factors influencing ASD neurobiology and diagnostic biomarkers. Factors such as demographic characteristics, clinical heterogeneity, and imaging protocols may contribute to these variations, highlighting the need for personalized and context-specific approaches in ASD diagnosis and treatment.

## 4 Conclusion

In conclusion, our proposed algorithm represents a significant advancement in the early detection and diagnosis of Autism Spectrum Disorder (ASD) using functional MRI (fMRI) data. By addressing the challenges associated with identifying ASD based on brain functional connectivity (FC), our approach offers several key advantages over existing methods. Firstly, the integration of wavelet transformation enables the extraction of temporal features from regions of interest (ROIs), allowing for the capture of spatiotemporal characteristics inherent in BOLD signals. Secondly, our approach incorporates an averaging strategy on the FC matrix and employs thresholding to eliminate spurious connections below a predefined threshold. Thirdly, the utilization of attention mechanisms enables the identification of influential connections within the brain network, highlighting nodes that play critical roles in ASD pathology. Lastly, our adoption of a multi-scale perspective, achieved through the extraction of features from two graph convolutional layers and their subsequent concatenation, provides a comprehensive representation of node features. By demonstrating superior performance compared to recent studies and offering high interpretability, our approach holds promise for improving the quality of life for individuals affected by ASD and advancing our understanding of its neurobiological underpinnings. Moving forward, continued research and validation efforts are warranted to further refine and validate our algorithm for real-world clinical applications.

## Data availability statement

The datasets presented in this study can be found in online repositories. The names of the repository/repositories and accession number(s) can be found in the article/supplementary material.

## Author contributions

CW: Writing – original draft. ZX: Methodology, Writing – review & editing. YX: Investigation, Writing – review & editing. QZ: Software, Writing – review & editing. JC: Software, Writing – review & editing.
